# Quick biochemical markers for assessment of quality control of intraoperative cell salvage: a prospective observational study

**DOI:** 10.1186/1749-8090-9-86

**Published:** 2014-05-14

**Authors:** Peng Dong, Ji Che, Xiuliang Li, Ming Tian, Fang Gao Smith

**Affiliations:** 1 Department of Anaesthesiology, Beijing Friendship Hospital, Capital Medical University, No. 95, Yong’an Road, Xicheng District, 100050 Beijing, China; 2 Perioperative, Critical Care and Trauma Trials Group, University of Birmingham, Edgbaston B15 2WB, Birmingham, UK

**Keywords:** Cell salvage, Clearance rate, Free haemoglobin, Albumin, Calcium

## Abstract

**Background:**

Intraoperative Cell Salvage (ICS), hereby referred to ‘mechanical red cell salvage’, has been widely used in adult elective major surgeries to reduce requirement for homologous red blood cell transfusion and its associated complications. However, amount of free haemoglobin (fHb) from ICS has been shown related to incidence of renal failure. fHb is the most important indicator of quality control of cell salvaged blood, thus monitoring the fHb concentration is imperative to minimise renal injury. However, currently there has been lacking quick biochemical markers to monitor the levels of fHb during ICS. The aim of this study was to screen quick biochemical markers for evaluating the amount of fHb during use of intraoperative cell salvage.

**Methods:**

Twenty patients undergoing elective cardiovascular surgery were enrolled. Blood was collected and processed using a Fresenius continuous auto-transfusion system device. The concentration of fHb, albumin (Alb), and calcium (Ca) in three washing modes were measured, and their clearance rates were calculated. The correlations among the clearances and concentrations of fHb, albumin, and calcium were analysed.

**Results:**

In three washing modes, concentrations of albumin and calcium are significantly associated with amount of fHb:fHb(g/L) = 0.111Alb(g/L) –0.108, R = 0.638, p = 0.000; fHb(g/L) = 1.721Ca(mmol/L) +0.091, R = 0.514, p = 0.000. Furthermore, the clearance rates of albumin and calcium significantly predict clearance of fHb, CR_fHb_ = 0.310CR_ALB_ + 0.686, R = 0.753, p = 0.000, CR_fHb_ = 0.073 CR _Ca_ + 0.913, R = 0.497, p = 0.000.

**Conclusions:**

In clinic practice, clearance rates of albumin, or calcium can be used to evaluate the quality of salvaged blood, fHb. Bed-side measurement of calcium could offer a more feasible means for clinicians to undertake a real-time assessment of fHb.

## Background

In the surgeries involving major blood loss, allogeneic red blood cell transfusion is often life-saving. However, allogeneic red blood cell transfusion has been shown to be associated with short-term and long-term complications of infectious diseases, non-infectious serious hazards of transfusion (NISHOTs) [1], or increased mortality [2-4]. This has led to the development of intraoperative cell salvage (ICS) and autologous red blood cell transfusion as important and effective blood conservation methods to reduce or avoid the need for homologous red blood cell transfusion and its associated complications.

ICS has now been widely used, as a substitution for allogeneic red blood cell transfusion, in adult elective major surgeries [5-12]. Over the years, ICS has been shown to be safe and efficacious to reduce the amount of allogeneic red blood cell transfusion required [13]. Thus, the research focus on ICS has moved to the issue about how to ensure the best quality of salvaged blood to be transfused back to the patient. High concentration of free haemoglobin (fHb) during ICS can result in haemoglobinuria that is associated with increased incidence of renal failure [14]. fHb is the most important indicator for quality cell salvaged blood, thus monitoring the fHb concentration is imperative to minimise renal injury. However, the measurement of fHb is not a routine laboratory test and also time consuming, which hampers improvement of quality control of cell salvaged blood. Albumin (ALB) and calcium (Ca) mainly exist in the plasma, the destroyed RBC during cell salvage has less impact on concentration of albumin and calcium. The molecular weight of albumin and calcium, the same as fHb, are lighter than erythrocyte, they should be eliminated together with the fHb during the procedure of centrifuging, so the clearance rates of these two should be expected to be similar with fHb. Therefore, the aim of this study was to assess whether quick biochemical markers, albumin and calcium, that can be used clinically to reflect the clearance of fHb for evaluating the quality control of salvaged blood.

## Methods

### Study participants

The institutional Ethics Committee of the Beijing Friendship Hospital approved the study proposal. Written informed consent was obtained from all participants. Adult patients undergoing elective cardiovascular surgeries with an estimated intraoperative blood loss of more than 800 ml were enrolled in this study. Exclusion criteria included the patients with haematopathy, malignancy, a contaminated operative field, or requirement for allogeneic red blood cell transfusion during current surgery.

### Cell salvage procedure

As we previously reported [15], Fresenius Continuous Auto Transfusion System device (CATS, Germany) was used for this study. The standard operative procedures were performed according to the manufacturer’s recommended protocols. Trained executive operators performed the blood salvage process. Intraoperative shed blood was collected through a special suction tube for blood collection. The vacuum pressure of the suction was maintained below 0.02 MPa to minimise haemolysis by suction. Blood was heparinized with physiological saline (30000 IU heparin in 1000 mL of 0.9% saline). The initial speed of the anticoagulant was 60 drops/min, which was continuously delivered to the tip of the suction catheter. The aspirated blood was filtered and stored in the blood reservoir. When enough blood was collected, the cell-washing device was operated according to the manufacturer’s recommended parameters. As the blood enters the processing chamber, the chamber is spinning at a speed of 1400–2400 RPM to generate centrifugal force. Separation of salvaged blood components mainly depends on the balance between densities of the various constituents of blood. Because red cells are heavier than other blood components, they will stay against the walls of the chamber while the smaller, lighter particles will be closer to the core of the chamber. In the following washing phase, RBCs are resuspended with 0.9% saline and blood plasma is further removed. At last, the saline is removed and the RBCs are concentrated and emptied into a reinfusion bag, and when appropriate, reinfused into the patient. CATS designed several washing programmes for different operations. Big flow is recommended for the operation with massive blood loss in a short time, standard mode is for the operation with normal blood loss speed, and high quality mode is for the shed blood with more debris.

### Blood sample collection

Three phases are involved in the cell salvage. During phase 1, blood is collected from the operating field to the reservoir using heparinised physical saline as the anticoagulant. During phase 2, three modes: big flux mode, standard mode and high quality mode, were used to separate, wash and concentrate the cells to reinfusion bags. In phase 3, concentrated washed red cells are then reinfused into the patient.

The blood samples were collected from the following time points and sites. The first time blood samples (3 ml) were obtained from venous blood. The second blood samples (10 ml) obtained from the reservoir following the blood in the reservoir was well mixed during phase 1. The third blood samples (5 ml ×3) were collected from the reinfusion bag after each mode was completed during phase 2. The volume of shed blood and the volume of reinfusion bag of each washing mode were recorded for calculating clearance rate. All blood samples were analysed within 20 min after collection.

### Analysis of blood samples

Free haemoglobin concentrations were measured using a HemoCue Plasma Low/Hb Photometer (HemoCue, Inc., Angelholm, Sweden). Haemoglobin (Hb) concentrations were measured using a HemoCueHb 201+ Analyser (Kuvettgatan 1, Angelholm, Sweden). Albumin concentrations were measured using HITACHI 7600 automatic biochemical analyser (Japan), and the concentration of calcium and haematocrit (Hct) were measured using GEM Premier 3000 blood gas machine (USA). The internal quality control of HITACHI 7600 automatic biochemical analyser was done with the standard samples from Wako Pure Chemical Industries every morning, and external quality control was done with the samples provided by National Centre for Clinical Laboratories every four months. The quality control of GEM Premier 3000 blood gas machine was done with the standard samples from Instrumentation Laboratory.

### Calculation of clearance rate

For the calculation of clearance rate, the concentrations of fHb, albumin and calcium in the reservoir and reinfusion bag, the volumes of shed blood in the reservoir for different washing modes, the corresponding volumes of washed blood in the reinfusion bag, the concentrations of Hb in the reinfusion bags of different washing modes were measured and recorded.

The efficacy of collection, centrifugation, and washing was assessed by the clearance rate (CR) of fHb, albumin and calcium, which were calculated using the following equations

$$\begin{array}{ll} \mathrm{Clearance}\;\mathrm{Rate}\;\left(\%\right)= & \left(1-\frac{\mathrm{Solut}e_{\mathrm{after}}\;\times \;\mathrm{Volum}e_{\mathrm{after}}}{\mathrm{Solut}e_{\mathrm{before}}\;\times \;\mathrm{Volum}e_{\mathrm{before}}}\right)\\ & \;\times \;100\% \end{array}$$

Where solute_after_ was the concentration of fHb, albumin or calcium of the washed blood in the reinfusion bag, volume_after_ was the volume of washed blood in the reinfusion bag, solute_before_ was the concentration of fHb, abumin or calcium of blood collected in the reservoir, and volume_before_ was the volume of blood collected in the reservoir.

The percentage of haemolysis was calculated using the following formula [14]:

$$\begin{array}{l} \mathrm{Percentage}\;\mathrm{of}\;\mathrm{haemolysis}\left(\%\right)\\ \;=\left[\left(\mathrm{fHb}/\mathrm{Hb}\right)\times \left(1‒\mathrm{Hct}\right)\right]\times 100\% \end{array}$$

Where fHb, Hb and Hct were the concentrations of fHb, Hb and haematocrit (Hct) in the reinfusion bag.

### Statistical analysis

All statistical analyses were performed using SPSS 13.0 software (SPSS, Inc., Chicago, IL, USA). Descriptive statistics were calculated on all measures to determine the characteristics of the sample, to check normality assumptions and to ensure adequate variability. All results are presented as mean ± standard deviation (SD) or incidence (n,%). Univariate linear regression analysis was applied to quantify the association of the clearance rates of fHb, albumin and calcium. A P-value of 0.05 was considered to be statistically significant.

## Results

From March to November 2012, 20 patients (13 males) undergoing off-pump Coronary Artery Bypass Grafting surgeries (n = 13) and vascular surgeries with an American Society of Anaesthesiology physical status II-IIIwere enrolled to the study. The mean age of the patients was 60.0 ± 14.0 years old (49–81 years old). The mean preoperative concentration of albumin was 38.3 ± 3.1 g/L (31.0-42.4 g/L), and the mean of calcium was 2.20 ± 0.09 mmol/L (2.09-2.44 mmol/L), which were in the normal range. The concentration of albumin and calcium were 18.4 ± 5.1 g/L (9.8-31.4 g/L) and 0.61 ± 0.17 mmol/L (0.33-0.99 mmol/L) in the reserviors, 4.6 ± 1.6 g/L (1.2-8.4 g/L) and 0.19 ± 0.08 mmol/L (0.10-0.37 mmol/L) in the reinfusion bags. After the procedure of cell salvage, the concentration of fHb, clearance rate of fHb and the percentage of haemolysis see Table 1.

**Table 1:** Concentration of fHb, clearance rate of fHb and percentage of haemolysis of washed blood

**n = 20**	**Washed blood**
Concentration of fHb	0.40 ± 0.27 g/L
Clearance rate of fHb	97% ± 2%
Percentage of haemolysis	0.21% ± 0.11%

### Correlation between concentrations fHb and albumin

**Figure 1:**
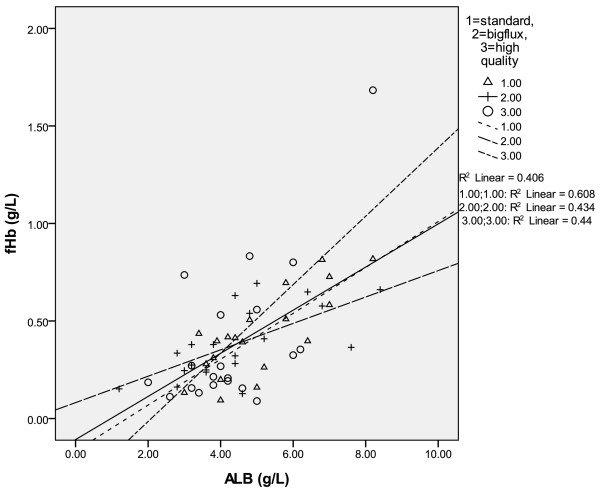
In three washing modes, the correlation between fHb and albumin.

In each of these three modes, (big flux, standard and high quality modes) there existed positive correlation between the concentration of albumin and that of fHb in the reinfusion bag. In the mode of big flow, fHb(g/L) = 0.118ALB(g/L) –0.166, r = 0.780, p = 0.000; in the standard mode, fHb(g/L) = 0.068ALB(g/L)-0.082, r = 0.659, p = 0.002; in the high quality mode, fHb(g/L) = 0.175ALB(g/L) –0.366, r = 0.663, p = 0.001. In combined three washing modes, the correlation between fHb and albumin in the reinfusing bag was fHb (g/L) = 0.111ALB(g/L) –0.108, r = 0.638, p = 0.000 (see Table 2, Figure 1).

**Table 2:** The results of the univariate regression analyses between fHb (g/L) and albumin (ALB, g/L)

**Washing mode**	**Regression analysis between fHb and albumin**	**r**	**p**
Big flow	fHb = 0.118ALB -0.166	0.780	0.000
Standard mode	fHb =0.068ALB-0.082	0.659	0.002
High quality mode	fHb =0.175ALB -0.366	0.663	0.001
Combined three modes	fHb =0.111ALB -0.108	0.638	0.000

### Correlation between concentrations fHb and calcium

**Figure 2:**
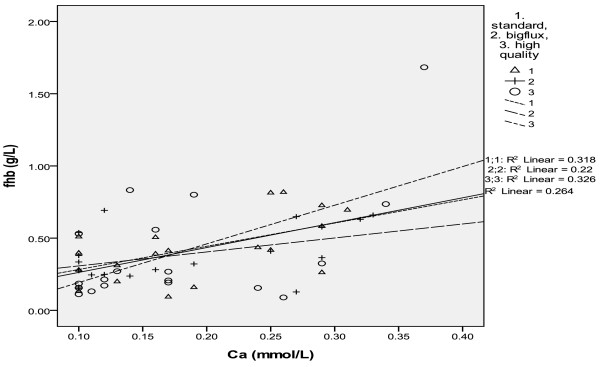
In three washing modes, the correlation between fHb and calcium.

In three modes, there existed correlation between the concentration of calcium and that of fHb in the reinfusion bag. In the mode of big flow, fHb(g/L) = 1.608Ca(mmol/L) + 0.122, r = 0.564, p = 0.010; in the standard mode, fHb(g/L) =0.967Ca(mmol/L) + 0.211, r = 0.469, p = 0.037; in the high quality mode, fHb(g/L) = 2.676Ca(mmol/L) –0.075, r = 0.571, p = 0.011. In combined three washing modes, the correlation between fHb and calcium in the reinfusing bag was fHb(g/L) =1.721Ca(mmol/L) + 0.091, r = 0.514, p = 0.000 (see Table 3, Figure 2).

**Table 3:** The results of the univariate regression analyses between fHb(g/L) and calcium(Ca, mmol/L)

**Washing mode**	**Regression analysis between fHb and Ca**	**r**	**p**
Big flow	fHb = 1.608Ca + 0.122	0.564	0.010
standard mode	fHb =0.967Ca + 0.211	0.469	0.037
High quality mode	fHb =2.676Ca -0.075	0.571	0.011
Combined three modes	fHb =1.721Ca + 0.091	0.514	0.000

### Regression relationship between clearance of fHb and clearance of albumin

**Figure 3:**
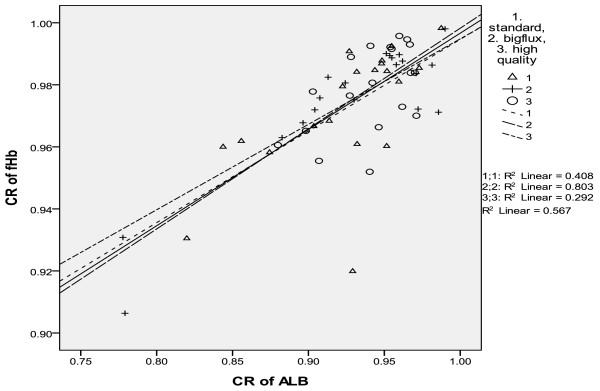
In three washing modes, the relationship of the clearance rate of fHb and that of albumin.

In the three modes of washing, there existed univariate regression relationship between the clearance rate of fHb(CR_fHb_ ) and that of albumin(CR_ALB_). In the mode of big flow, CR_fHb_ = 0.295CR_ALB_ + 0.699, r = 0.638, p = 0.008; In the standard mode, CR_fHb_ = 0.324CR_ALB_ + 0.675, r = 0.896, p = 0.000;In the high quality mode, CR_fHb_ = 0.275CR_ALB_ + 0.719, r = 0.540, p = 0.017. In combined three washing modes, the regression relationship of clearance of fHb and clearance of albumin in the reinfusing bag was CR_fHb_ = 0.310CR_ALB_ + 0.686, r = 0.753, p = 0.000 (See Table 4, Figure 3).

**Table 4:** **The results of the univariate regression analyses between CR**_**fHb **_**and CR**_**ALB**_

**Washing mode**	**Regression analysis between CR**_**fHb **_**and CR**_**ALB**_	**r**	**p**
Big flow	CR_fHb_ = 0.295CR_ALB_ + 0.699	0.638	0.008
Standard mode	CR_fHb_ = 0.324CR_ALB_ + 0.675	0.896	0.000
High quality mode	CR_fHb_ = 0.275CR_ALB_ + 0.719	0.540	0.017
Combined three modes	CR_fHb_ = 0.310CR_ALB_ + 0.686	0.753	0.000

### Regression relationship between clearance of fHb and clearance of calcium

**Figure 4:**
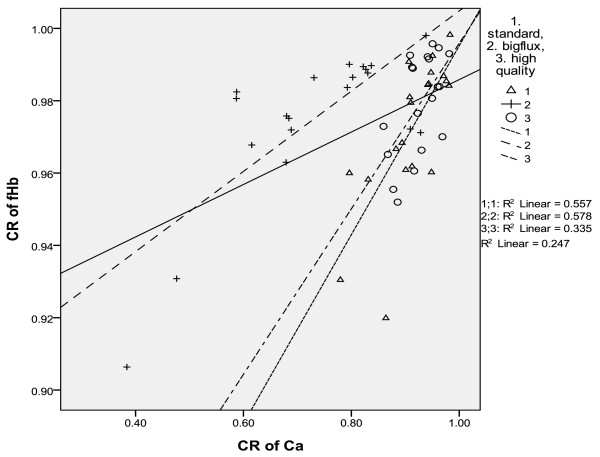
In three washing modes, the relationship of the clearance rate of fHb and that of calcium.

Under three modes of washing, there was linear regression relationship between the clearance rate of fHb and that of calcium(CR_Ca_). In the mode of big flow, CR_fHb_ = 0.261 CR_Ca_ + 0.734, r = 0.746, p = 0.000;In the standard mode, CR_fHb_ = 0.111CR_Ca_ + 0.894, r = 0.760, p = 0.000; In the high quality mode, CR_fHb_ = -0.100CR_Ca_ + 0.997, r = 0.592, p = 0.008. In combined three washing modes, the regression relationship between clearance of fHb and clearance of calcium was CR_fHb_ = 0.073 CR _Ca_ + 0.913, r = 0.497, p = 0.000 (see Table 5, Figure 4).

**Table 5:** **The results of the univariate regression analyses between CR**_**fHb **_**and CR**_**Ca**_

**Washing mode**	**Regression analysis between CR**_**fHb **_**and CR**_**Ca**_	**r**	**p**
Big flow	CRfHb = 0.261 CRCa + 0.734	0.746	0.000
Standard mode	CRfHb = 0.111CRCa + 0.894	0.760	0.000
High quality mode	CRfHb = -0.100CRCa + 0.997	0.592	0.008
Combined three modes	CRfHb = 0.073 CR Ca + 0.913	0.497	0.000

## Discussion

Thus far, there has been no information on the quality standards of fHb for both mechanical red cell salvaged blood from autologous red blood cell transfusion and for stored blood from allogeneic red blood cell transfusion. The quality standard level of haemolysis rate has been referred to stored blood < 1% in the USA or < 0.8% in the Europe [16] as there has been no such information available for salvaged blood. Most studies regard salvaged blood with more than 90% clearance rate of fHb as good quality control [12,15]. Our study results, produced under good quality control of intraoperative cell salvage (0.21% haemolysis rates and 97% clearance rate of fHb), should be generalizable.

During the process of cell salvage, red blood cells (RBC) may be destroyed by the suction with negative pressure and centrifugation. These destroyed RBC will increase fHb. When the fHb level in plasma is > 1 g/L, the metabolic load may exceed the tolerance in adults, and methaemoglobin may form to cause organ injury. In particular, circulating fHb may precipitate within renal tubules, which leads to haemoglobinuria nephropathy and tubular dysfunction [14]. However, fHb > 4 g/L frequently occurs in washed RBC following severe haemolysis. Thus, it is crucial to monitor fHb concentrations to assess salvaged blood quality in order to prevent patients from renal and other organ injury.

However, in clinical settings, measurements of concentrations of fHb have to be undertaken in a specialised haematological laboratory, which usually takes more than two hours before the results become available. This is useless for clinicians to be able to manipulate the process of cell salvage to improve the quality of cell salvaged blood before reinfusion.Bed-side fHb analyser is currently available to be able to assay the concentration of fHb accurately in 60 seconds. But the instrument is too expensive to be applied widely.

The basic mechanism of autotransfusion system device is to eliminate the components that the molecular weight lighter than that of erythrocyte through the centrifuging. The molecular weight of albumin and fHb are 66000 Dalton and 67000 Dalton respectively, lighter than erythrocyte. Molecules of Alb and fHb should stay at the same layer and be eliminated together during the procedure of centrifuging, so the clearance rates of these two should be expected to be similar. Our study suggests there is a linear relationship between the concentrations of fHb and albumin. Therefore, the regression equation will allow us to calculate the concentration of fHb from the concentrations of albumin. Also, clearance rate of fHb is positively correlated with clearance of albumin and their correlation coefficient *r* 0.638, 0.896 and 0.540 for three washing modes (big flux, standard or high quality) respectively. This association again has offered clinicians a feasible mean to learn the clearance rate of fHb from the clearance rate of albumin. However, like lactate dehydrogenase that was strongly correlated to fHb [17], assays of albumin concentrations can only be undertaken in a biochemical laboratory although this is easier and less expensive than assays of fHb, the measurements have to be one-off or intermittent without a real time monitoring using a blood gas machine.

Potassium assay was suggested as a biochemical marker for evaluating the clearance of fHb, but the elimination of fHb during the washing process was not correlated with the residual concentration of potassium [17,18]. This finding may be explained by the fact that intracellular compartment of the red blood cells contains very high concentration of potassium and so degree of increase in fHb concentrations was not proportional to degree of increase in potassium concentrations when the RBC were destroyed. In a similar way, the elimination of fHb was not correlated to elimination of potassium. In contrast, calcium mainly exists in the plasma, the destroyed RBC during cell salvage has less impact on concentration of calcium. The molecular weight of calcium is lighter than the erythrocyte, so calcium, like fHb, is eliminated by centrifugation. Our study has proven that the clearance rate of fHb can be predicted by the known clearance of calcium, the latter can be calculated if the concentrations of calcium in the reservoir and reinfusion bag are measured. In the clinic practice, the concentrations of calcium can be measured in several minutes using a bed-side blood gas machine.

Either heparin or citrate can be used for anticoagulation during red blood cell salvage. There existed controversy about which anticoagulant is best. Also heparin anticoagulation increased the fHb level compared to citrate, but the haemolysis rate was still in normal range [19]. Because of its low price and ready availability, heparin is most commonly used [20].

Our study has limitations, firstly, the fHb concentrations of patients’ blood were not tested before and after the transfusion of cell salvaged red blood cells; secondly, the study has a small sample size in cardiovascular surgery only. These findings are promising and further studies with larger sample size in a number of types of surgeries are needed to confirm these outcomes and to establish whether these clinical biochemical markers will offer utility in this regard.

## Conclusion

In summary, during the use of Continuous Auto Transfusion System device for cardiac and vascular surgeries, the concentration and clearance rate of albumin, and calcium can be employed as useful biochemical markers to evaluate the quality control of cell salvaged blood, fHb, in the washing modes of big flux, standard and high quality. Because the concentration of calcium can be assayed quickly using a blood gas machine in the operation room, bed-side measurement of calcium could offer a more feasible means for clinicians to undertake a real-time assessment of fHb.

## Competing interests

The authors declare that they have no competing interests.

## Authors’ contributions

MT had the concept, PD designed and set up the study with supervision from MT and FGS. PD, JC and XL obtained the consents, collected all the samples and undertook the measurements. PD completed analysing the data with statistical support from a statistician and drafted the manuscript. FGS was chiefly responsible for the acquisition and interpretation of data and for finalising the manuscript. MT, FGS and PD also were involved in critical review of the manuscript. PD completed revision after submission. All authors read and approved the final manuscript.
